# Exploring the Complexities of Atopic Dermatitis: Pathophysiological Mechanisms and Therapeutic Approaches

**DOI:** 10.26502/jbb.2642-91280155

**Published:** 2024-07-17

**Authors:** Fihr Chaudhary, Wismmy Lee, Tony Escander, Devendra K Agrawal

**Affiliations:** Department of Translational Research, College of Osteopathic Medicine of the Pacific, Western University of Health Sciences, Pomona CA 91766, USA

**Keywords:** Allergic march, Alopecia areata, Atopic dermatitis, Calcineurin inhibitor, Colloidal oatmeal, Environmental factors, JAK inhibitor, Microbiome, Moisturizer, Oxidative stress, Phototherapy, PDE4 inhibitor, Skin barrier dysfunction, Topical corticosteroid, Wet wrap therapy

## Abstract

Atopic dermatitis (AD) is a prevalent inflammatory skin condition impacting both children and adults globally, with a prevalence of 15–30%. It ranks as the most prevalent skin disorder based on disability-adjusted life-years by the World Health Organization. It presents with symptoms like skin irritation, redness, dryness, itchiness, and vesicular blisters and commonly coexists with other atopic symptoms like allergic rhinitis, asthma, and food allergies. The pathophysiology involves a complex interplay of genetic predispositions, immunological dysfunctions, and environmental factors leading to tissue inflammation and disrupted skin barrier integrity. Alopecia areata is characterized by nonscarring hair loss and shares correlations with AD including a higher prevalence of atopic diseases, shared intracellular mechanisms involving the JAK-STAT pathway, and potential treatment overlap such as dupilumab. These correlations could direct new areas of research and increased insight for both diseases. Treatment of AD requires a personalized approach due to its complex, multifactorial nature integrating nonpharmacological interventions like skin hydration and trigger avoidance as well as topical and systemic approaches, if necessary, with topical corticosteroids being the first line for flares; long term corticosteroid use poses risk for adverse effects like skin atrophy. Severe cases may require systemic treatments or phototherapy. Future treatment prospects include targeting the dysbiotic microbiome and identifying biomarkers for tailored therapeutic strategies, emphasizing the importance of personalized medicine in optimizing AD management.

## Introduction to Atopic Dermatitis

The prevalence of atopic dermatitis (AD) in both children and adults is from 15% to 30%. This illness is prevalent worldwide, with varying rates in different regions. An intricate interplay occurs among anomalies in the skin barrier function, environmental and viral agents, and immunological abnormalities, resulting in the onset of AD. Reactive oxygen species have been partially studied in Atopic Dermatitis and other skin conditions, although their involvement in Atopic Dermatitis has been seldom researched. This section aims to provide an overview of the epidemiology of Atopic Dermatitis.

Atopic dermatitis (AD) is a complex, long-lasting inflammatory skin condition that is often associated with other atopic symptoms. These manifestations include of food allergy, allergic rhinitis, and asthma. Atopic dermatitis impacts individuals of all ages, with prevalence rates varying from 15% to 30% [1] and ranked as the most prevalent skin disorder [2]. Dermatitis and atopy, collectively referred to as atopic dermatitis (AD), constitute the two components of the acronym AD. Atopy is a term introduced by Coca and Cooke in 1923. It describes any reaction mediated by IgE, including reactions that are suitable and proportionate to the antigen. Although interaction with the allergen is necessary before hypersensitive reactions develop, there may be a genetic factor at play [3]. Atopic dermatitis may present as asymptomatic hypersensitivity to one or more atopic conditions such as allergic rhinitis, fever, and asthma. Dermatitis is a medical condition marked by skin redness, swelling, irritation, and the development of tiny blisters. Dermatitis is the result of skin irritation by an external substance or an allergic reaction.

Over time, different names have been proposed for the syndrome. One of these names is Prurigo Besnier, also referred to as Besnier’s itch. The condition was named after the French dermatologist Ernest Besnier (1831–1909). Atopic manifestations follow a specific sequence of events, starting with IgE antibody responses and leading to the development of clinical symptoms in early infancy. These symptoms might remain for years or decades but may improve on their own as a person age [4]. The “allergic march” describes the progression of atopic manifestations, often known as the “atopic march.”

Atopic Dermatitis primarily develops due to a malfunctioning skin barrier, resulting in dry and itchy skin. Scratching exacerbates this issue due to mechanical damage. Research suggests both the innate immune system and the adaptive immune system play a role in the development of Atopic Dermatitis [5]. In recent years, research has increasingly shown that oxidative stress (OS) has a role in Atopic Dermatitis (AD). Furthermore, it is widely known that oxidative stress induces tissue inflammation by suppressing the expression of genes involved in the synthesis of proinflammatory cytokines. When activated, inflammatory cells release free radicals. Due to its substantial inflammatory properties, it is plausible that oxidative stress could have a role in the onset of Atopic Dermatitis [6].

### Epidemiology

In both children and adults, the prevalence of Atopic Dermatitis (AD) is estimated to be between 15 and 30 percent, and the incidence has increased by two to three times over the course of the last few decades in industrialized countries [1]. According to the Global Burden of Disease survey conducted by the World Health Organization (WHO) in 2010, AD was placed #1 among common skin disorders in terms of disability-adjusted life-years and years lived with a disease [2]. To evaluate the epidemiology and regional heterogeneity in the prevalence of Atopic Dermatitis, a study that was carried out in three phases as part of an international investigation that included one million participants was carried out [2]. In different parts of the world, the prevalence of AD and the region with the highest prevalence level continue to change. Latin America has emerged as a region with relatively high prevalence in follow-up data [7]. Previously, the places with the highest incidence were Nigeria, the United Kingdom, and New Zealand. However, in recent years, the prevalence has increased in Latin America. A significant rise in the incidence of atopic diseases has also been observed in a few international emerging nations. Over the course of the past four decades, there has been a rise in the number of cases of Atopic Dermatitis in India. The most prevalent form of dermatosis among children who were registered at a pediatric dermatology clinic was age-related dermatosis (AD), which accounted for 28.46% of all patients who were registered. According to a study report published by the Postgraduate Institute of Medical Education and Research in Chandigarh, India, the prevalence of Atopic Dermatitis was found in 210 newborns (up to one year) and children (n = 462) over various seasons [8].

### Pathogenesis of Atopic Dermatitis

Atopic Dermatitis (AD) pathophysiology has been a mystery to researchers for decades. There are still a great number of undiscovered points that need to be found to put forward a notion that is all-encompassing, despite the fact that significant milestones have been accomplished in the process of describing the mechanisms that precipitate Atopic Dermatitis in persons who are genetically predisposed to the disease. According to Alsaad, the histological manifestation of recurrent itching and dermo-epidermal inflammation is characterized by hypertrophy of the dermis and epidermis, along with the presence of eosinophils, macrophages, and T lymphocytes [9]. Furthermore, the inflammatory response in Atopic Dermatitis starts when an IgE-associated Langerhans cell in the skin attaches an antigen and then transmits the antigen to T cells. This results in the release of many cytokines, including a variety of IL. These mediators cause an increase in the number of inflammatory cells that are recruited, which ultimately results in eczema [10]. The development of Atopic Dermatitis is thought to be multifaceted, with complicated interactions between susceptibility genes, immaturity and/or anomalies in barrier function, and environmental variables [11]. In general, the relationship between these components is complex. Deficiencies in the function of the skin barrier, inflammation, immune system, and ocular surface are all intertwined in a complicated manner. Researchers have not yet fully investigated the role that reactive oxygen species play in Atopic Dermatitis (AD), even though it is thought that these species do play a part in the disease [12]. The term “oxidative stress” (OS) refers to a state in which there is an imbalance between the production of free radicals and the antioxidant defense mechanism that is present in the body. For the past fifteen years, its implications in Atopic Dermatitis have been documented in scientific literature [13]. The inflammation of the skin, which is a hallmark of Atopic Dermatitis, is made worse by obstructive sleep apnea. It has been shown that OS has a role in the activation of NFκB pathways, which in turn increases the expression of genes and, ultimately, the manufacture of antioxidant enzymes [12]. However, activation of the NFκB pathway also leads to the production of proinflammatory cytokines, such as IL-6, IL-8, IL-9, and IL-33. This, in turn, leads to an increase in the dermal inflammatory infiltrate and the release of histamines in the skin, which further exacerbates the symptoms [14]. Even in animals that do not have atopic dermatitis, OS has been shown to be responsible for itching and scratching, according to a few investigations conducted on animals. Itching can be caused by the repeated application of chemicals like formaldehyde or using intradermal hydrogen peroxide [15]. This can occur through the increased expression of IL-4 or through the histamine-independent pathway, respectively.

Through the process of lipid oxidation, OS causes damage to the epidermal keratinocytes by causing disruptions in the DNA, the cellular enzymes, and/or the cell membrane structures and molecules. These changes within the cells themselves are exhibited histomorphologically as epidermal oedema or spongiosis, as well as a disruption in the stratum corneum. Ceramides are among the most essential lipids that play a role in contributing to the preservation of a healthy skin barrier. It is during the process of keratinization in the stratum corneum that sphingosine and fatty acids are formed, which are the components that make up these substances. The presence of an epidermal barrier that is not damaged serves to restrict the entry of allergens and other infectious agents, which in turn helps to reduce the loss of water through the transdermal layer. According to the findings of a few research [16], the epidermal barrier is directly affected by oxidative stress (OS) that is caused by contaminants from the environment. To trigger inflammation of the skin, environmental contaminants like cigarette smoke attach to aryl hydrocarbon receptors, which then causes the creation of reactive oxygen species, DNA damage, and the generation of inflammatory cytokines. On the other hand, certain flavonoids could bind to aryl hydrocarbon receptors, which eventually leads to the activation of nuclear factor erythroid 2-related factor-2 (Nrf2), which in turn produces essential molecules that shield cells from the damaging effects of oxidative stress [17].

Microorganisms found on the skin could be another source of OS. A genetic predisposition, poor immunity, epidermal barrier dysfunction, and environmental factors have been suggested to have a close link in the etiology and pathogenesis of Atopic Dermatitis [18]. These four primary components are involved in the development of Atopic Dermatitis.

It was proven that urine markers of OS, including as 8-hydroxydeoxyguanosine (8- OHdG), nitrite or nitrate, and selenium, are altered in children who have Atopic Dermatitis [19]. The levels of these markers are significantly higher in children who have Atopic Dermatitis compared to children who do not have the condition. An increased oxygen saturation (OS) and an altered balance of oxygen and nitrogen radicals have been hypothesized to have a role in the pathogenesis of Atopic Dermatitis in children. Preschool children with Atopic Dermatitis had malondialdehyde levels that were much higher and blood antioxidant capacity that was significantly lower than those of controls [20]. In more recent times, there have been out case-control research on individuals who suffer from eczema, using healthy persons as controls [25]. The research discovered that patients with eczema had much higher levels of lipid peroxidation, which was evaluated by measuring blood malondialdehyde, and lower amounts of antioxidants, including vitamins A, C, and E [21]. This was in comparison to the control group, which had significantly lower levels of these antioxidants. In patients with alopecia areata, an inflammatory skin disorder that is closely associated to Atopic Dermatitis [22], similar findings of the presence of OS and enhanced lipid peroxidation were revealed. Following that, it has been discovered oxidative stress and impaired antioxidant defenses in children who had acute onset of Atopic Dermatitis [23, 24]. During the hospitalization period, they discovered that the levels of bilirubin oxidative metabolites and urine glycosylation end products were much greater in children with Atopic Dermatitis [24].

### Genetic Factors

There is a possibility that food allergens are the primary cause of Atopic Dermatitis (AD) in the early stages of life. However, after that, environmental aeroallergens become more significant and may relate to developing respiratory sensitization. There is a lack of clarity regarding the mode of inheritance and the genes that are involved [25]. To keep the integrity of the skin barrier intact, it is essential to have a protein called filaggrin. This protein is responsible for binding to keratins that are related with keratinocyte development. The production of filaggrin is reduced because of genetic abnormalities that induce failure in the skin barrier and loss of water through the trans epidermal space, both of which are the root causes of eczema. This results in a greater penetration of allergens into the skin, which in turn causes allergic sensitization, asthma, and hay fever [26].

### Skin Barrier Dysfunction

In addition to its role as a primary defense mechanism, the epidermis also performs the function of a biosensor at the surface of the skin. As a main mechanism for the development of Atopic Dermatitis (AD), skin barrier deficiencies are now regarded to be a primary contributor. These defects allow infections, allergens, and other environmental insults (toxins, irritants, and pollutants) to easily enter the body.30 % In Atopic Dermatitis (AD), the function of the epidermal barrier is damaged due to various abnormalities that are responsible for the barrier failure. One of these abnormalities is a reduction in lipids, specifically ceramide and sphingosine reduction. In clinical settings, the disruption of the skin barrier function that occurs in atopic skin results in an increase in the loss of water through the trans epidermal space as well as an increase in the penetration of allergens, irritants, and microorganisms [27].

### Immunological Responses

A biphasic inflammation is a characteristic of the immune response that is present in Atopic Dermatitis. In the initial and acute phase of Atopic Dermatitis (AD), a Th2-based immune response (IL-4, IL-13, thymic stromal lymphopoietin, and eosinophils) is typically observed. However, in chronic AD skin lesions, a Th1/Th0 dominance has been described (IFN-γ, IL-12, IL-5, and granulocyte-macrophage colony-stimulating factor) [28]. During Atopic Dermatitis, cytokines and chemokines are important variables. Leukocytes, particularly monocytes, from atopic patients exhibit enhanced phosphodiesterase activity [29]. This results in decreased levels of cAMP and increased production of prostaglandin and IL-10, both of which block the function of Th1 cells and stimulate the production of IgE.

### Host and Environmental Factors

Early in life, incidences of severe atopic dermatitis have been associated with the existence of food sensitization and allergy. Approximately 50–70% of children who develop AD at a young age are allergic to one or more substances, including cow’s milk, hen’s eggs, and peanuts, as well as home dust mite, pollen, and pets. Food allergies are prevalent in children with AD, with a suggested link ranging from 20% to 80%, however the more often acknowledged percentage is 30% [30].

### Relationship with Alopecia Areata

In addition to being characterized by nonscarring hair loss, alopecia areata (AA) is a prevalent dermatological illness that affects up to 2% of the population [31]. Clinical manifestations can range from limited and patchy hair loss to *alopecia totalis*, which refers to the complete loss of hair on the scalp, or alopecia universalis, which comprises the complete loss of hair on both the scalp and the body [32]. There have been a few preliminary epidemiological studies and meta-analyses that have shown a higher prevalence of atopic diseases in patients with AA. These diseases include atopic dermatitis (AD), asthma, and allergic rhinitis (which is typically caused by Th2 skewing), which suggests that these conditions share an immunological background [33]. Finally, the treatment arsenal that is currently accessible for patients who have atopic illnesses, such as Atopic Dermatitis (AD), is fast developing [34]. As a result, it is essential to have a deeper understanding of the correlations that exist between these conditions and AA.

The condition known as alopecia areata (AA) is an autoimmune illness that is tissue- specific and cell-mediated. As far as the cytokine balance is concerned, AA has been classified as a type 1 inflammatory disease. In contrast, atopic dermatitis (AD), which is a type 2 inflammatory condition, is frequently more difficult in regard to the underlying pathogenesis compared to AA. The immunological status of AA may be distinct between patients with atopic dermatitis and patients without atopic dermatitis, as well as between patients with extrinsic and intrinsic Atopic Dermatitis [35]. This study provides a significant idea: type 2 immunity may play a role in the development of Atopic Dermatitis (AA) in people who have extrinsic Atopic Dermatitis [36]. It is possible to argue that the immunological condition of atopic dermatitis (AA) that is not atopic is distinct from that of atopic AA. Contact dermatitis, mental health issues, and autoimmune illnesses are the next most common conditions in AA patients, followed by atopic diathesis, which includes allergic rhinitis, asthma, and/or eczema. The prevalence of atopic diathesis in AA patients can reach as high as 38.2% per patient. According to the findings of seven studies that involved cross-sectional research, the prevalence of atopic dermatitis and atopic history in adults ranges from 22 percent to 38 percent when confounding variables are considered [35]. Atopic Dermatitis is largely a Th2-driven disease that is characterized by elevated levels of interleukin (IL)-4, IL-5, IL-13, and IL-31 [37].

Among the innovative medicines for atopic dermatitis (AD), dupilumab is a completely human antibody that identifies IL-4Rα and inhibits the signaling pathways of both IL-4 and IL-13 receptors. The JAK-STAT pathway is the system that is downstream of these receptors [38]. Recent findings have demonstrated that dupilumab is useful as a treatment agent not only for Atopic Dermatitis (AD), but also for Alopecia Universalis [39]. This finding raises the hypothesis that the inflammatory components of these two diseases are mysteriously connected to one another. The development of Atopic Dermatitis and Atopic Dermatitis is regulated by a shared intracellular mechanism that involves JAKs and the signaling pathways connected with them [40]. The JAK-signal transducer and activator of transcription (JAK-STAT) pathway is utilized by cytokines, such as interleukin-1 (IL) and interferon-alpha (IFN-α), to successfully regulate gene expression by transmitting signals from the cell membrane to the nucleus [41]. JAK-STAT-dependent cytokines, including interferon-gamma (IFN-γ) and interleukin-15 (IL-15), have a role in promoting the activation and proliferation of autoreactive T-cells in autoimmune alopecia (AA). Similarly, the JAK-STAT signaling pathway is utilized by IL-4, IL-5, and IL-13 to induce Th2 immunity in Atopic Dermatitis.

In total, 9.4% of individuals diagnosed with Atopic Dermatitis had a history of Alopecia Areata (AA), either in the past or in the present [42]. Accordingly, the prevalence of Atopic Dermatitis was shown to be greater in research where individuals with Atopic Dermatitis self-reported having the condition than in trials where the disease was diagnosed by a physician [42]. AA patients had higher probabilities of Atopic Dermatitis (AD) than control patients who did not have AA, according to the pooled analysis of the three trials that included control patients who did not have Atopic Dermatitis (AA) [42]. Overall, those who suffer from alopecia areata, particularly alopecia totalis or alopecia universalis, are at a greatly elevated risk for Atopic Dermatitis.

### Current Treatments

**Figure 1:**
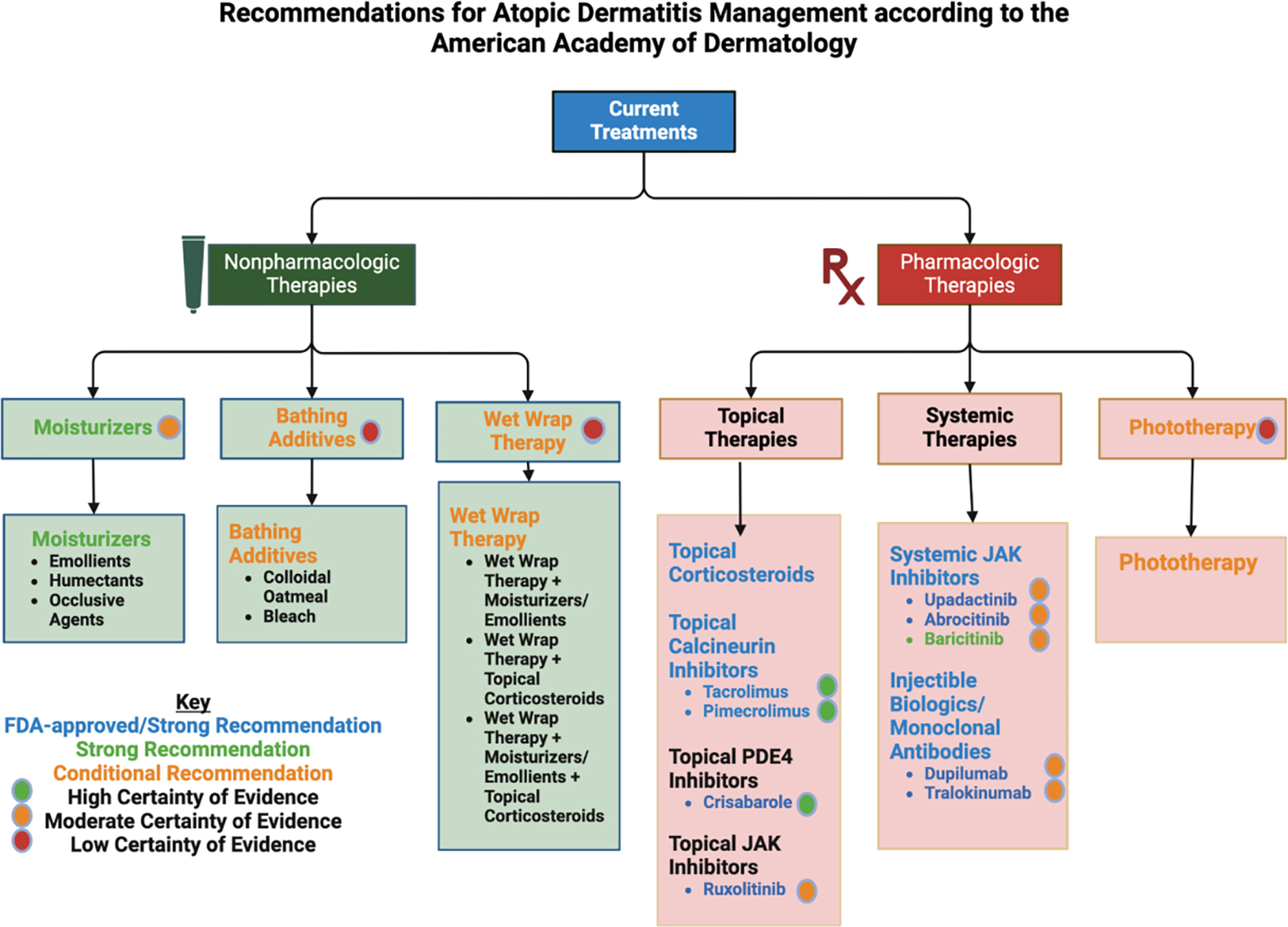
Recommendations for Atopic Dermatitis Management according to the American Academy of Dermatology. This chart compiles information regarding recommendation and certainty of evidence of current treatments for Atopic Dermatitis according to the American Academy of Dermatology as of 2023. This chart is based on the “Figure 1: Adults with atopic dermatitis” of “Guidelines of care for the management of atopic dermatitis in adults with phototherapy and systemic therapies” [46] but adds on to it by providing evidence certainty based on “Guidelines of care for the management of atopic dermatitis in adults with topical therapies” [6]. Created with BioRender.com

Due to the nature of atopic dermatitis (AD) being a complex skin condition with a multitude of factors from genetic, immunological, and environmental, an effective treatment strategy requires a personalized approach for the various maladies of the disease such as skin xerosis/dryness, pruritus/itch, and infection as there currently exists no cure for AD [43]. No matter the severity, patients with mild to severe forms of AD may receive some benefit from basic nonpharmacological interventions such as skin hydration, bathing modifications, wet wrap therapy and elimination of common triggers and allergies [44]. However, these strategies are typically paired in conjunction with topical medications as part of a comprehensive treatment plan [44]. For patients with severe AD that is “not responsive to topical treatments”, systemic treatments and phototherapy may be necessitated [45]. Here in, we explore nonpharmacological therapies, topical medications, systemic treatments, and phototherapy, as well as emerging/potential treatments for AD. A summary of the recommendations by the American Academy of Dermatology for AD management is shown in Figure 1.

### Nonpharmacologic Treatments Moisturizers

**Figure 2:**
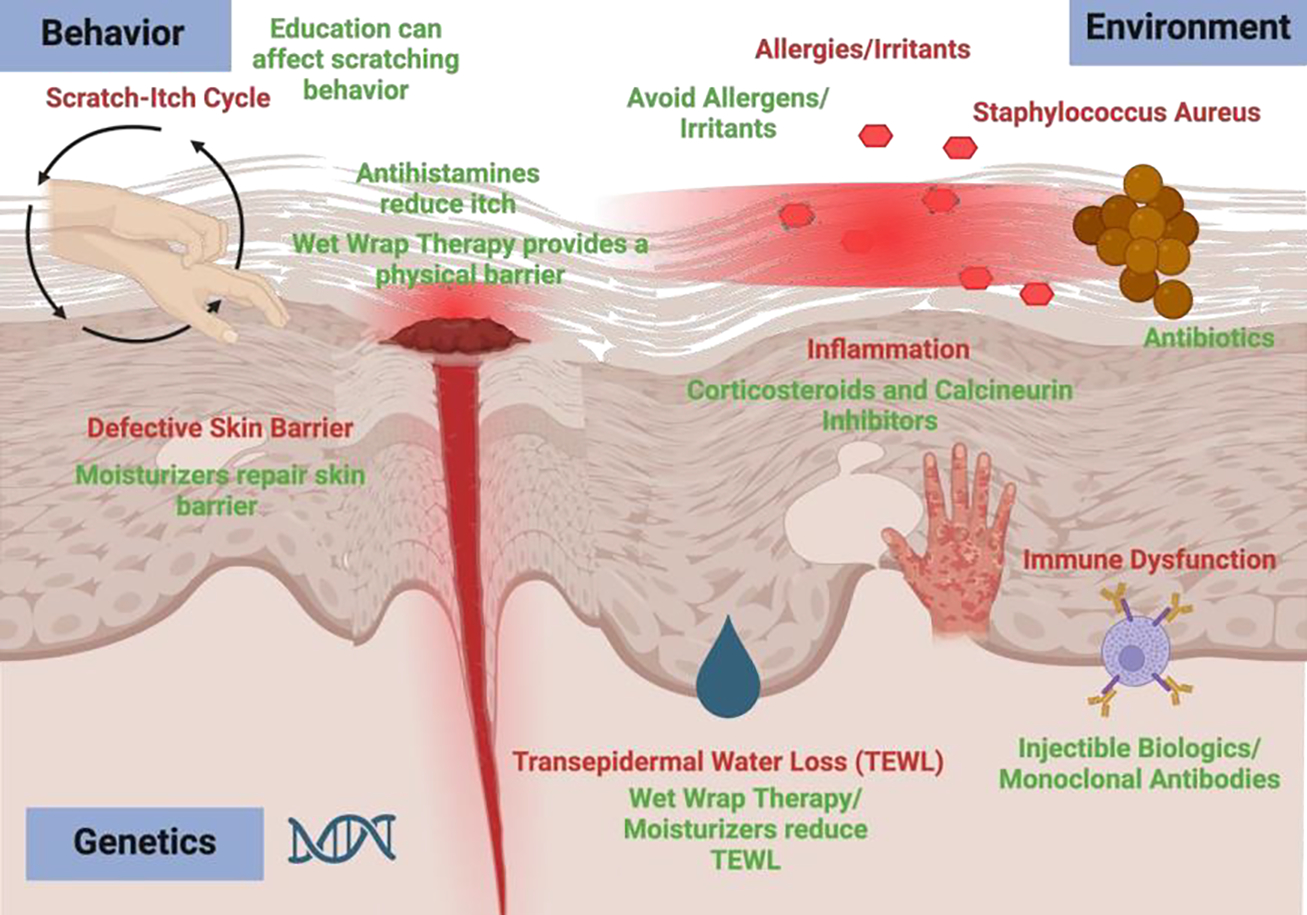
Contributing Factors of Atopic Dermatitis and Treatment Options. There are multifactorial causes of atopic dermatitis. Inflammation can cause itch that can cause scratching which can cause tissue damage. Defective skin barriers can also be caused by genetics/epigenetics and lead to transepidermal water loss (TEWL). Moisturizers maintain skin hydration necessary for skin barrier repair. Wet wrap therapy can also prevent TEWL by providing an occlusive barrier as well as a physical barrier that prevents scratching. Inflammation can lead to itch which can be decreased with antihistamines. Itching leads to scratching. Scratching can also be reduced with education to change patient behavior. Allergens and irritants can penetrate a dysfunctional skin barrier leading to irritation and inflammation and the need for avoidance of allergens/irritants. Inflammation can be reduced with pharmacologic such as topical corticosteroids and calcineurin inhibitors. Dysbiosis microbiomes, especially ones overran with staphylococcus aureus can be reduced using antibiotics and lead to skin barrier defects. Created with Biorender.com.

The most basic recommendation for management of mild to severe AD is a liberal and frequent application of moisturizer [44]. According to the American Academy of Dermatology Association, moisturizers are “strongly recommended” with “moderate strength of evidence” to reduce the severity of disease symptoms for AD [45, 46]. A systematic review of 5 studies totaling 488 participants (treatment group: 279, control group: 209) revealed a standardized mean difference of −0.51 representing a moderate effect on symptom severity reduction [45, 46]. By maintaining hydration, moisturizers reduce pruritus and aid in the repair of skin barrier defects [45, 46]. Composed of ingredients like occlusive agents, humectants, and emollients, moisturizers prevent trans- epidermal water loss (TEWL), attract water, and soften skin [47]. The formulation needed depends on the severity of skin dryness with emollients (glycol, glyceryl stearate, etc.) and humectants (hyaluronic acid, urea, etc.) usually being sufficient for normal to dry skin while occlusive agents (petrolatum, mineral oil, etc.) may be required for dry to inflamed skin [48]. Although moisturizers are shown to be generally safe, they can also present a risk of adverse effects (Figure 2). A systematic review of 5 studies including 545 participants showed 34.3% (117/341) in the treatment group experience mild adverse effects compared to 22.1% (45/204) of the control group which shows an increased risk of adverse effects (RR: 1.32, 95% CI: 1.01–1.74) [45]. Thus, it is important to carefully examine the ingredients as some moisturizers may contain irritating or potentially allergenic ingredients [45].

### Colloidal Oatmeal

Skin actives like colloidal oatmeal can also be added to moisturizers or baths for their therapeutic purposes. Colloidal oatmeal is a U.S. Food and Drug Association approved skin protectant treatment for atopic dermatitis due to its anti-pruritic, anti-inflammatory and skin repairing properties [48]. Scratching an itch, one of the most common and aggravating symptoms of AD, can lead to increased pruritus and inflammation according to the “itch-scratch” cycle [49]. This leads to skin epithelium damage which can trigger keratinocytes to release pro-inflammatory cytokines like MCP-1 which induces IL-6 expression and inflammatory/immune responses characteristic with AD [49]. Colloidal oatmeal contains avenanthramides which are compounds shown to suppress Interleukin-6 (IL-6), Interleukin-8 (IL-8) and Monocyte chemoattractant protein-1 (MCP-1). By decreasing pro-inflammatory cytokines, colloidal oatmeal reduces inflammation as well as itch caused by cytokine release [50]. Colloidal oatmeal has also been shown to aid in skin barrier repair [51]. In a clinical study of 61 participants with mild to moderate eczema (sample size of 30 in treatment group: sample size of 31 in the control group), those who received a 1% colloidal oatmeal eczema cream showed an average reduction of 54% in Atopic Dermatitis Severity Index (ADSI) and 51% in Eczema Area and Severity Index (EASI) scores and increased skin barrier repair compared to a standard moisturizer in just 14 days [51]. The 1% colloidal oatmeal eczema cream was also associated with a decrease in staphylococcus aureus (S. aureus) (which commonly colonizes AD skin and contributes to skin barrier dysfunction). Along with improved microbiome composition, colloidal oatmeal can also improve skin barrier function by promoting a normal acidic skin pH and providing a protective and occlusive barrier due to fatty acids and lipids found in colloidal oatmeal [50].

### Bathing

Along with colloidal oatmeal, diluted bleach has been commonly added to baths to treat atopic dermatitis. Chronic AD is associated with persistent bacterial infections, especially since AD skin lesions have increased susceptibility to S. aureus colonization [42]. Thus, in theory, diluting bleach baths with antiseptic and antimicrobial effects may assist in preventing bacterial infection [45]. However, a systematic review and meta- analysis of 4 randomized control trials (RCT) composed of 116 patients revealed that although both bleach bath and water-only bath groups showed a reduction in AD severity from baseline, there was no statistically significant difference in disease severity or staphylococcal colonization between treatment and control groups, suggesting that bleach baths are not necessarily more effective than water baths alone. Emollients are another common bath additive which are thought to leave a protective barrier over the skin, decreasing TEWL [52] (Figure 2). Like bleach baths however, a study composed of 483 children with AD compared the benefits of emollient bath additives vs. water alone for 52 weeks and found no statistically significant difference between treatment or control conditions [52]. There currently exists no standard for duration/frequency of bathing, bathing additive, or water temperature for AD due to limited evidence which suggests new avenues for potential research [45]. One study however suggests that more frequent bathing followed by the application of occlusive moisturizers (soak and seal) is preferred for AD management. RCT assigned 40 children with moderate to severe AD to 2 groups [53]. Group 1 had twice weekly baths for less than 10 minutes for the first 2 weeks followed by twice daily baths for 15–20 minutes for the subsequent two weeks while Group 2 did the reverse [53]. The results revealed a statistically significant 30% reduction in SCORAD score with more frequent bathing compared to less frequent [53]. Another study attempts to give evidence to the common belief that lukewarm rather than hot water should be used, however it is limited using healthy volunteers, rather than patients with AD. One study cold water increased TEWL (control: 25.75 g.h^−1^.m^−2^, treatment: 34.96 g.h^−1^.m^−2^, p<0.001), but had no statistically significant difference in erythema compared to control, and that hot water increased skin erythema (control: 249.45 AU, treatment: 286.34 AU, p<0.001) and TEWL (control: 25.75 g.h^−1^.m^−2^, treatment: 58.58 g.h^−1^.m^−2^, p < 0.001) compared to baseline [54]. When comparing cold water vs hot water, there was statistically significant difference in erythema (cold water: 253.63 AU, hot water: 286.34 AU, p < 0.001) and TEWL (cold water: 34.96 g.h^−1^. m^−2^, hot water: 58.58 g.h^−1^.m^−2^, p < 0.001) between cold water and hot water conditions [54]. Although this suggests that cold water is preferred over hot water for the prevention of TEWL and erythema, more research is needed to prove if this cold-water recommendation will also be parallel for patients with AD [54].

### Wet Wrap Therapy

Wet wrap therapy (WWT) is when a layer of topical emollient, topical corticosteroid (TCS) or both is used directly on the skin under a moist layer of gauze/bandages and an external dry layer which can be applied for periods of 1 hour to 2 weeks [45]. The multiple layers provide scratch-protection which prevents the perpetuation of the itch-scratch cycle as well as an occlusive barrier that results in increased absorbance and decreased TEWL [45]. This treatment has conditional recommendation from the AAD with low certainty [45]. A 14-day study of 24 acute AD patients compared corticosteroid treatment with prednicarbate and WWT on one limb and prednicarbate-only treatment on another limb. A statistically significant improved SCORAD score in WWT and prednicarbate group was seen compared to the prednicarbate only group, however the mild to moderate difference may not be considered clinically significant (MD: 1.4, 95% CI: −2.75, −0.05) [45]. It is important to note that no withdrawals/adverse effects were observed in either group giving evidence that WWT is a relatively safe addition to basic AD management [45]. However, a systematic meta-analysis of 6 trials (sample sizes: 19 to 51 participants) which compared WWT and TCS and TCS without WT and showed a statistically insignificant increased risk of mild skin infections with the addition of WWT [55]. American Academy of Dermatology conditionally recommends the use of WWT with low certainty of evidence [45]. Despite this, due to its relatively safe nature, WWT can be an option for those who want a barrier against scratches and an extra boost in hydration, however it has drawbacks such as increased time and effort and education needed for WWT and that the benefit of WWT has mostly been observed in pediatric populations [45].

### Food Allergies/Trigger Avoidance

Atopic dermatitis is part of the atopic triad; thus, patients with AD are associated with higher incidence of asthma and allergic disease as the conditions are closely linked [56]. A systematic review and meta-analysis revealed that food allergies were commonly seen in 32.7% of AD patients (4–5 times greater than healthy reference patient) and atopic dermatitis was seen in 45.3% of patients with food allergies with strongest association observed in patients with severe AD and children [57]. Elevated IgE levels are common in both AD and food allergies with common IgE triggering foods being eggs, milk, peanuts, wheat, etc. [56]. Thus, it has been theorized that elimination diets that remove chronic triggers or food allergies may have the potential to reduce the severity of AD. A systematic review and meta-analysis of 10 RCT of 599 participants with mild to moderate AD showed that dietary elimination may slightly improve eczema severity and SCORAD score compared with no dietary elimination (treatment group: 50%, control group: 41%) [58]. Care should be taken when experimenting with elimination diets for the treatment of AD to maintain a balanced diet without nutritional defects [56]. Furthermore, another concern is that the removal of chronic triggers can reduce one’s immune tolerance to that food which can cause increased sensitivity when/if reintroduction occurs [56].

### Pharmacologic Treatments

#### Topical Corticosteroids

**Figure 3:**
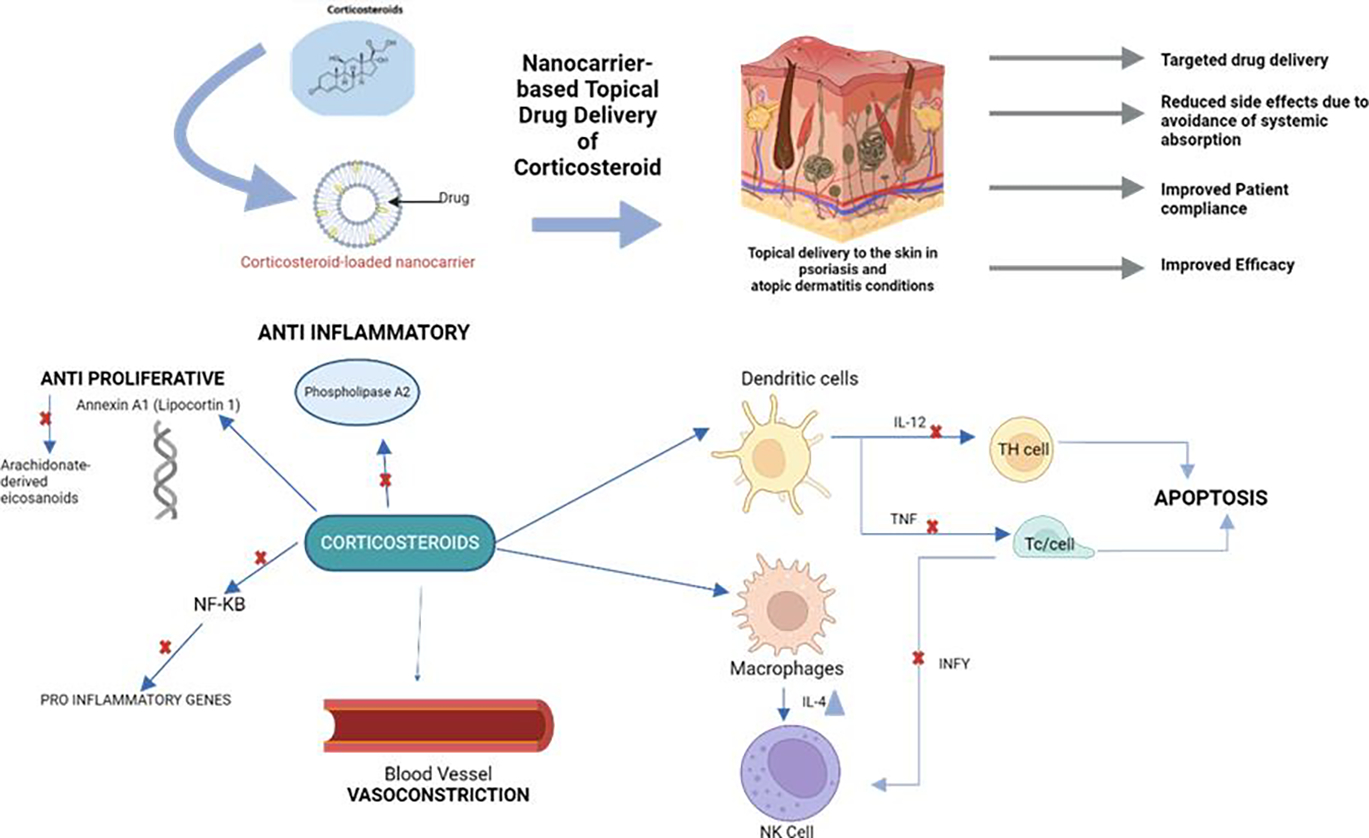
Mechanism of action of Topical Corticosteroids. Because of their anti- inflammatory, immunosuppressant, vasoconstrictive, and antiproliferative properties, corticosteroids are useful for a range of dermatological diseases. By attaching to phospholipids, blocking phospholipase A2 and eicosanoids, and reducing cell-mediated inflammation, they lessen the generation of inflammatory proteinoid. In addition to lowering local blood flow via vasoconstriction, they limit cytokine synthesis and decrease mast cell activation.

Topical corticosteroids (TCS) are FDA-approved to reduce inflammation, pruritus, and relapses and act as the first line of treatment for AD flares after basic management with moisturizers [45]. The underlying cellular and molecular mechanisms of the effect of topical corticosteroids are shown in Figure 3. A 2023 systematic review and meta- analysis by the American Academy of Allergy, Asthma & Immunology and American College of Allergy, Asthma, and Immunology reviewed 219 RCT (43,123 pediatric and adult participants with mild to moderate AD) and compared 68 topical AD treatments and their safety and efficacy on 7 factors ranging from disease and itch severity, quality of life, flares, sleep disturbance, and adverse effects [59]. Out of the 68 topical AD treatments ranging from TCS, wet wrap therapy, topical calcineurin inhibitors (TCI), PDE4 inhibitors, JAK inhibitors, etc., the study found that group 5 TCS which are lower to medium potency TCS was the most effective, improving 6/7 factors with “moderate-to-high certainty of evidence”

The effects of TCSs have also been seen in pediatric patients younger than the age of 2 years old which is important as AD disproportionately affects those in this age group [60]. A 2019 systematic review and meta-analysis composed of 12 RCT and 2224 participants found that 65% of participants responded to TCSs (95% CI, 0.54–0.74) compared to 32% of those in the control vehicle/moisturizer group (95% CI, 0.20–0.48) and found similar rates of adverse effects with 17% of participants having adverse events in TCS groups (95% CI, 0.08–0.33) compared to 12% in control (95% CI 0.02–0.42) [60]. TCS range from class I/high potency to class VII/very low potency groups [45]. Some dermatologists prefer to use the lowest potency needed to produce the desired effect as high potency formulations can have increased risk of adverse effects while others prefer a moderate-to-high strength TCS for acute management [61]. Low potency TCSs (0.25– 1% hydrocortisone, 0.1% dexamethasone, 0.01% fluocinolone acetonide, etc. and medium potency TCS (0.005–0.05% fluticasone propionate, 0.1% betamethasone valerate, 0.1% triamcinolone acetonide can be used for a longer duration with reduced atrophy risk compared to higher potencies [45]. High potency TCS (0.05% betamethasone dipropionate, 0.05% fluocinonide) and very high potency TCS (0.05% clobetasol propionate, 0.05% halobetasol propionate, etc.) are typically used to treat severe AD flares [45].

A lower potency of TCS should be used for the face, neck, and genitals than the rest of the body. The frequency of TCS applications typically ranges from twice weekly to 1–2 times daily for a two-week period [45].

The mechanism of action making TCS effective for AD flare treatment is its antimitotic, anti-inflammatory, and immunosuppressive effects [61]. However, TCS can present with cutaneous and systemic adverse effects with long-term use [44]. Due to its vasoconstrictive and antimitotic effects, TCSs have an increased risk of skin atrophy, higher potency TCSs, occlusion and in elderly populations [61]. Other cutaneous adverse effects include pigment alteration, delayed wound healing, purpura, and red face syndrome/red scrotum syndrome [44]. One of the most common systemic adverse effects of TCS is suppression of adrenals, especially with high-potency TCS used on large areas of the body or in various forms [45]. However, short term use of TCS may be relatively safe according to an umbrella review of 38 systematic reviews which found no statistically significant increased risk of skin thinning when TCSs were used twice weekly to prevent flares vs. control in 5 RCTs [62]. In the same umbrella review, a meta-analysis of 11 uncontrolled observation studies (522 participants) using any potency of TCS revealed 3.8% adrenal suppression of cortisol, however the effects were reversed when treatment was discontinued [62]. More studies are necessary to show the long-term effects of TCS use as such studies in the umbrella review composed of RCTs that were about 2 weeks long [62].

#### Topical Calcineurin Inhibitors

In the 2023 systematic review and meta-analysis by the American Academy of Allergy, Asthma & Immunology and American College of Allergy, Asthma, and Immunology, as mentioned above, topical calcineurin inhibitors (TCIs) such as 0.1% tacrolimus and 1% pimecrolimus were found to be one of the most effective topical AD treatments for children and adults, following closely after group 5 TCSs which improved 6/7 AD outcomes (most effective for 3 outcomes) with pimecrolimus improving 6/7 outcomes (most effective in 2 outcomes) and tacrolimus improving 5/7 outcomes (most effective in 2 outcomes) [59]. Thus, TCIs are effective anti-inflammatory treatment options for those with AD without the long-term secondary effects of TCSs [6]. A study of 413 AD patients had 210 patients apply 0.1% tacrolimus and 203 patients apply 1% pimecrolimus for 6 weeks and showed a reduction of mean EASI score of 54.1% in the tacrolimus group and 34.9% in the pimecrolimus group after 6 weeks (P = 0.0002) [45]. The AAD strongly recommends the use of TCIs such as 0.03%–0.1% tacrolimus and 1% pimecrolimus cream with a high certainty of evidence [45]. However, pimecrolimus may be a better option for patients who have milder AD or are at greater risk of irritation as one of the adverse effects of TCIs, especially tacrolimus, are itching/burning sensations [45]. A 2021 systematic review and meta-analysis composed of 4 cohort studies and 3,048,838 patients compared the incidence of lymphoma, Hodgkin lymphoma, and non-Hodgkin lymphoma patients treated with TCIs and showed a higher incidence of lymphoma (0.02– 0.09%) in TCI groups vs. control (0.02–0.06% [63]. Tacrolimus (RR 1.68, 95% CI: 1.39– 2.04) and pimecrolimus (RR 1.40, 95 % CI 1.13–1.74) both showed an increased risk of lymphoma, especially non-Hodgkin lymphoma (tacrolimus: RR 1.89; 95 % CI: 1.53–2.32 and pimecrolimus: RR 1.38; 95 % CI: 1.09–1.74) [63].

#### Topical PDE-4 inhibitor

**Figure 4:**
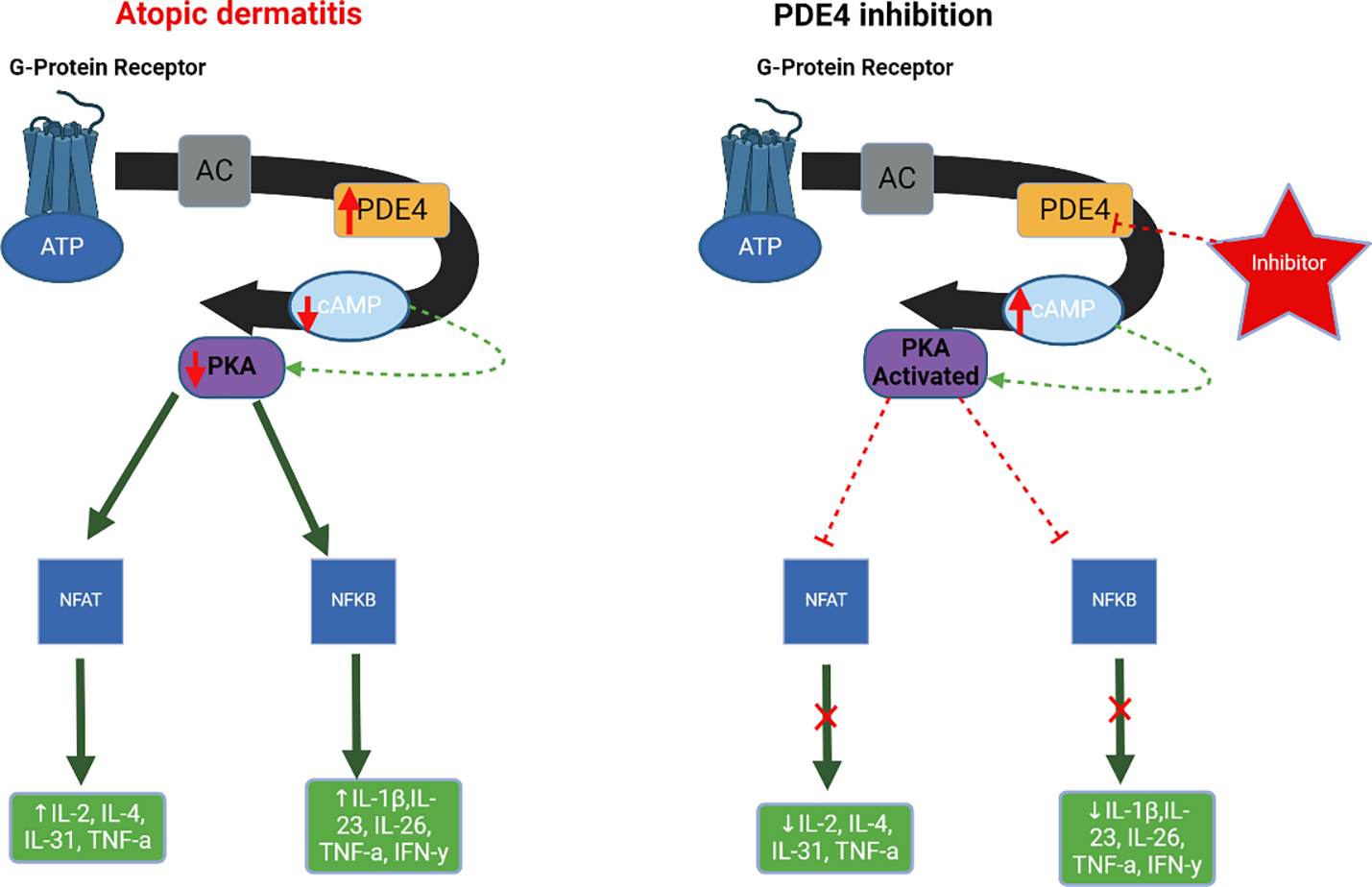
Atopic dermatitis is characterized by an increase in the activity of phosphodiesterase 4 (PDE4). PDE4 controls the synthesis of inflammatory substances by breaking down cyclic adenosine monophosphate (cAMP). Blocking PDE4 results in elevated levels of cAMP inside cells, which triggers the activation of protein kinase A (PKA). PKA activation suppresses the signaling pathways of NFAT and NFkB, as well as the release of downstream cytokines and chemokines. AC, Adenylyl cyclase; ATP, adenosine triphosphate; IL, interleukin; IFN-γ, interferon gamma; TNF-α, tumor necrosis factor-α.

Atopic dermatitis is characterized by an increase in the activity of phosphodiesterase 4 (PDE4) that breaks down cAMP and enhance the synthesis of inflammatory mediators (Figure 4). Inhibition of PDE4 will enhance cAMP and thus will inhibit pro-inflammatory cytokines. A topical PDE-4 inhibitor such as 2% crisaborole ointment is an FDA-approved non-steroidal and anti-inflammatory treatment for mild-to-moderate AD treatment that is strongly recommended by the AAD with high certainty of evidence [45]. AD patients typically present with an increase in phosphodiesterase 4 which contributes to an increased production of inflammatory cytokines like IL-4 and IL-13, thus using PDE-4 inhibitors may reduce the number of inflammatory cytokines and reduce the disease severity [64]. In 2 RCTs, patients from 2–79 years of age were given either 2% crisaborole treatment twice daily (1016 participants) or control group (506 participants) [45]. After 28 days, there was a statistically significant improvement in Investigator’s Static Global Assessment (IGA) with 326/1016 (32.1%) of treatment group vs 110 /506 (21.7%) of control group reaching clear/almost clear assessment (RR: 1.80, 95% CI: 1.48–2.18, P <.0001) [45]. In a 2019 meta-analysis reviewing 7 double-blind RCTs with 1869 mild-to- moderate AD participants compared topical PDE-4 inhibitors vs control [64]. Crisaborole showed a statistically significant decrease in lesions vs. control at 14 days (SMD: −0.59; 95% CI, −1.15 to −0.02; P = .04) and 28 days (SMD: −0.86; 95% CI, −1.44 to −0.28; P = .004) [64].

Adverse effects of PDE-4 typically involve burning, stinging, itching, pain, and redness to the site of application, worsening of AD, and infections such as yeast infections [64]. In three studies that included PDE-4 inhibitors such as E6005, OPA-15406, and crisaborole, a significantly higher rate adverse effects were associated with crisaborole (RR, 3.70; 95% CI, 1.59–8.61; P = .002) vs. control [64]. However, for all 3 PDE-4 inhibitors, there was no statistically significant difference in adverse effects vs. control (RR: 1.45; 95% CI, 0.52–4.09; P = .48). There was also a small rate of withdrawal seen in three studies of three PDE-4 inhibitors (E6005, OPA-15406, and crisaborole) with 19/1111 (1.7%) of those in all 3 PDE-4 groups withdrawing compared to 14/569 (2.5%) of control group which showed not statistically difference in withdrawal between PDE4 inhibitor groups vs. control (RR: 0.74; 95% CI, 0.37–1.48; P = .39) [64]. Thus, suggesting that PDE-4 is a relatively safe alternative to TCSs and TCIs for the treatment of AD [64].

#### Topical and Systemic JAK Inhibitors

**Figure 5:**
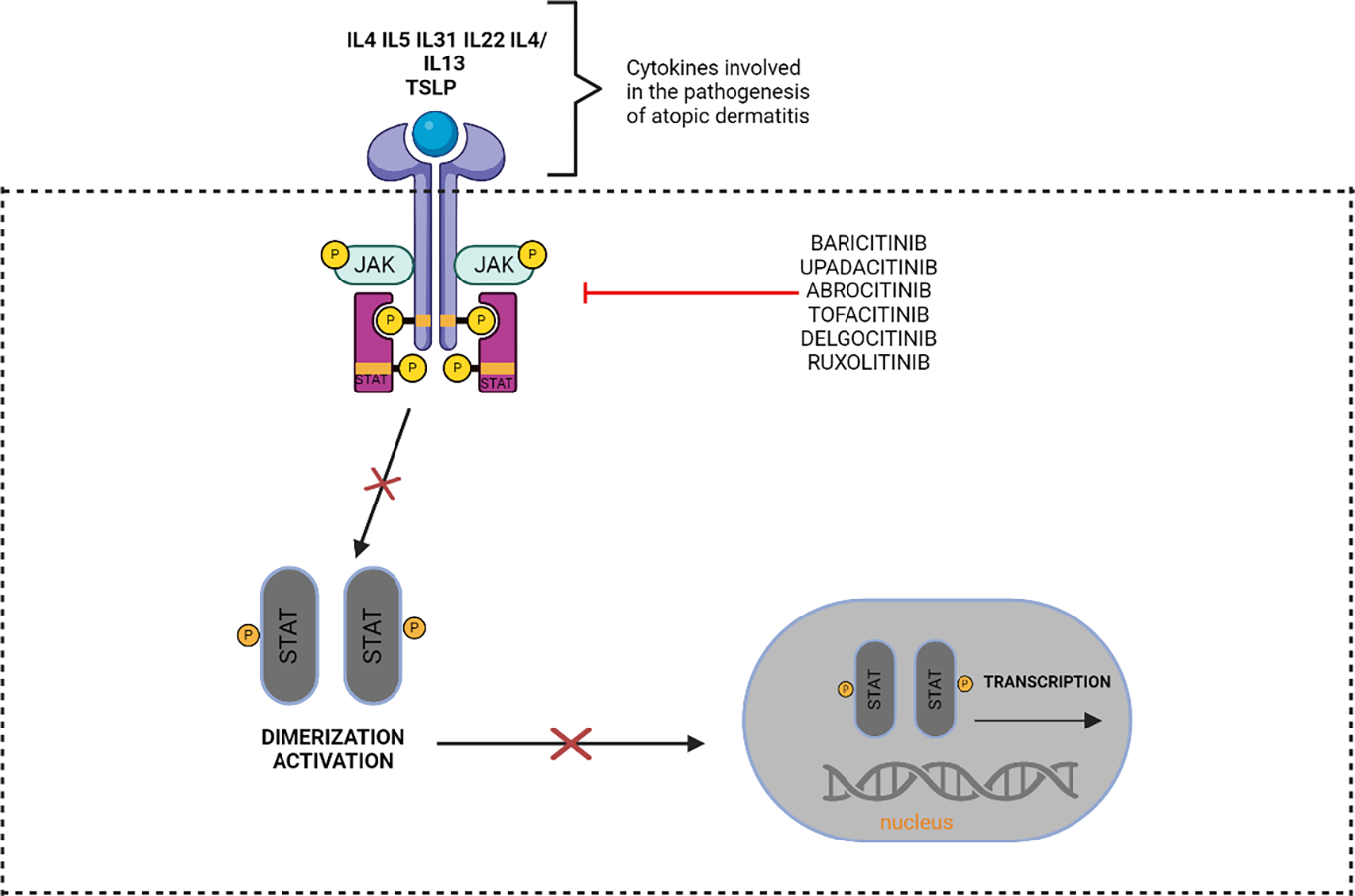
JAK/STAT and how the medications that suppress JAK work. When cytokines bind to their receptors, they trigger the phosphorylation of JAK and STAT proteins, which are needed for signal transduction through the JAK/STAT pathway. The second set of proteins regulate inflammatory factor production through dimerization and nuclear translocation. Preventing JAK phosphorylation and STAT activation is the main function of JAK inhibitors.

JAK inhibitors prevent JAK phosphorylation and STAT activation and thus could be beneficial in the treatment of AD (Figure 5). The topical JAK inhibitor ruxolitinib is an FDA- approved short-term treatment for mild to moderate AD for patients over the age of 12 years old [45]. Topical JAK inhibitors like 1.5% ruxolitinib reduces IL-4 and IL-13 cytokine signaling by inhibiting the JAK-STAT intracellular transduction pathway and the American Academy of Dermatology strongly recommends topical JAK inhibitors for the treatment of AD, however limited long-term safety data is available [45]. In 2 RCTs, 277/531 (52.2%) adult AD patients treated with ruxolitinib showed an IGA score of clear to almost clear compared to 33/296 (11.1%) of control (RR: 4.60, 95% CI: 3.05–6.95) [45].

Two oral systemic JAK inhibitors, upadacitinib and abrocitinib are also FDA-approved for the treatment of moderate to severe AD that does not respond to other systemic treatments like injectable biologics [65]. In comparison to injectable biologics, systemic JAK inhibitors “have predictable pharmacokinetics, do not elicit immunogenicity, allow for flexible dosing, and an oral mode of therapy” [65]. Upadacitinib has two approved doses at 15 mg and 30 mg with dose-dependent effects [65]. In 3 RCT with about 2500 moderate to severe AD participants 12 years or older, 60.1%–69.6% in the 15 mg group and 72.9– 79.7% in the 30 mg group reached EASI-75 which is a 75% improvement from baseline [65]. Furthermore, 38.8%–48.1% of the 15 mg group and 52%–62% of 30 mg group reached IGA 0/1 (clear/almost clear skin) vs. control at 16 weeks [65]. A 24-week phase 3b trial compared the efficacy and safety of 30 mg Upadacitinib and the injectable biologic dupilumab and saw an EASI-75 of 43.7% for Upadacitinib vs 17.5% for control at 2 weeks and an EASI-75 of 64.2% and 59.5% respectively at 24 weeks, suggesting that Upadacitinib is a treatment with quick-acting potential [65]. However, care should be taken when prescribing JAK inhibitors as they may potentially have an increased risk of thromboembolism, cardiovascular events, blood clots, cancer, and death [45].

### Systemic Treatments

#### Injectable Biologics/Monoclonal Antibodies

Dupilumab is an injectable biologic and monoclonal antibody that targets the IL-4 receptor and is an FDA-approved first-line systematic AD treatment for adults. One double RCT compared EASI and SCORAD scores for patients using 300 mg of dupilumab weekly vs. control at 16 weeks and showed a mean difference of −55.7% for EASI and - 43.1% SCORAD score improvement [66]. In a placebo-controlled, double-blind trial, the blood and skin biopsies of 54 moderate to severe AD patients were evaluated after randomization to 200 mg dupilumab treatment weekly vs. control after 16 weeks [67]. The skin biopsy results revealed that dupilumab was associated with a statistically significant decreased lesional epidermal thickness at 16 weeks compared to control (P = .0002) [67]. Dupilumab also showed a significantly improved expression of genes associated with lesional and non-lesional skin of 110.8% in dupilumab treatment groups vs. 55% in control at 16 weeks [67]. Dupilumab showed reduced expression of type II inflammatory genes like IL-13, IL-31, etc. [67]. The most common adverse effects of dupilumab were injection site reactions, conjunctivitis, and headache [66].

#### Phototherapy

Phototherapy, particularly narrowband ultraviolet-B radiation (NB-UVB) (313 nm wavelength) is a systemic treatment for moderate to severe AD when TCS and other systemic treatments have not succeeded [46]. A Cochrane review of 32 trials (1219 participants, 5–83 years old) reviewed phototherapy safety and efficacy for narrowband UVB (NB-UVB), broadband UVB, and UVA1, with 13 trials evaluating NB-UVB [68]. Analysis suggested that NB-UVB may have improved physician ratings, patient reported symptoms and IGA after 12 weeks vs control [68]. However, there is high risk of bias due to missing data, inappropriate analysis, or insufficient information [68]. In a study of 41 participants, there was a mean difference of −9.4 in reduction of physician assessment with NB-UVB vs control after 12 weeks (95% CI: −3.62 to −15.18). However, two trials composed of 37 participants reported little to no difference between NB-UVB vs. control after 4 to 6 weeks. Thus, the AAD conditionally recommends phototherapy with limited certainty of evidence [46].

Some concerns associated with phototherapy use are increased risk of skin cancer, sunburns, and heat intolerance [46]. Another consideration is that many phototherapy regimens require 2 to 3 treatments weekly for 10–14 weeks to be done at medical spas/treatment centers and typically require high insurance co-pays which can present a financial and time hardship on patients [46]. In two trials of NB-UVB (71 participants for 8 to 12 weeks of treatment, each trial reported one withdrawal per treatment group vs. control [68]. Thus, no statistically significant difference in adverse-effect related withdrawals between treatment vs. control has yet been found [68].

#### Emerging/Potential Treatments

There are many exciting ideas for possible treatments for AD. One idea involves targeting the dysbiosis microbiome that is typically characteristic of AD [69]. Microbiome transplants and topical probiotics with beneficial bacteria could hold potential in reducing the colonization of harmful bacteria, such as S. aureus which is associated with bacterial infections in AD [69]. Further research, however, is needed to test the efficacy of targeted microbiome treatments as topical probiotics needs to be done to test the efficacy of this as a treatment for AD as oral probiotics currently have limited success for the treatment of AD [69]. Endocannabinoids have also been explored as a potential treatment for atopic dermatitis, particularly CB1 agonists which have potential to reduce inflammation by suppressing mast cell activation in AD mouse models [70]. Topical CB1R agonist, α- oleoyl oleyl amine serinol, showed a statistically significant recovery of epidermal permeability barrier (p < 0.01) and reduced epidermal hyperplasia in an AD mouse model whereas mice who lacked CB1R showed decreased epidermal barrier recovery and increased ear swelling [70]. Further research on endocannabinoids is needed to assess their treatment potential for AD [70].

Since AD displays complex and multifactorial etiology, much of AD treatment is going towards the direction of personalized medicine. Biomarkers is an area of personalized medicine which will allow patients to avoid the current “trial-and-error” approach and instead rely on genetic, cytokine, immune, or microbiome biomarkers to identify patients who may be more likely to respond to a certain type of medication [69]. A Biomarkers in Atopic dermatitis and Psoriasis (BIOMAP) project looked at 56 articles and evaluated the 146 biomarkers that are associated with AD [71]. Filaggrin loss of function mutations contribute to skin barrier defects and was one of the most frequently investigated biomarkers, however showed limited association of AD comorbidity [71].

## Conclusion

In conclusion, atopic dermatitis (AD) and its pathogenesis involves a complex interplay of immunological dysregulation, environmental factors, and genetic predisposition. Its increasing incidence and widespread global prevalence underscore the need for effective management strategies. The immune system and skin barrier dysfunction play central roles in AD development, emphasizing the importance of interventions targeted to modulate immune responses and restore barrier function. While current treatments primarily focus on topical and systemic symptom management such as moisturization, topical corticosteroids, etc., emerging research directions offer promising avenues for more personalized therapies. Innovative approaches such as exploring the microbiome or biomarkers associated with genetic and immunological factors hold potential for revolutionizing AD treatment paradigms. Additionally, the correlations between other inflammatory conditions like alopecia areata (AA) which have a shared intracellular mechanism involving JAKs and signaling pathways may suggest opportunities for new directions of research and understanding. However, challenges remain in translating these scientific advancements into clinical practice, such as the development of standardized protocols for personalized medicine approaches and the need for rigorous testing of novel interventions. Furthermore, addressing the multifactorial and complex nature of AD requires interdisciplinary collaboration among dermatologists, immunologists, researchers, and allergists to optimize patient outcomes and improve quality of life for individuals living with AD.
